# Exploring age-related iron dysregulation: effects on longevity, body size, and behavior in *C. elegans*

**DOI:** 10.1016/j.exger.2025.112826

**Published:** 2025-07-03

**Authors:** Rola S. Zeidan, Pearl Ebea-Ugwuanyi, Shannon Sykes, Natalia Evripidou, Evelyn Pan, Zachary Markovich, Yi Sheng, Sung Min Han, Christiaan Leeuwenburgh, Rui Xiao

**Affiliations:** a Department of Physiology and Aging, College of Medicine, University of Florida, Gainesville, FL 32610, USA; b Department of Health Outcomes and Biomedical Informatics, College of Medicine, University of Florida, Gainesville, FL, USA; c Institute on Aging, University of Florida, Gainesville, FL 32610, USA; d Florida Chemical Senses Institute, University of Florida, Gainesville, FL 32610, USA; e Genetics Institute, University of Florida, Gainesville, FL 32610, USA; f UF Health Cancer Center, University of Florida, Gainesville, FL 32610, USA

**Keywords:** Iron, Aging, Mobility, Behavior, Mechanosensation, *C. elegans*

## Abstract

Age-related iron accumulation is widely observed in various species and significantly impacts physiological processes. However, systematic investigation into how age-related iron dysregulation affects different life traits is still limited. This study utilizes the model organism *C. elegans* to examine the roles of iron regulatory genes throughout different life stages, focusing on their effects on iron homeostasis, longevity, mobility, size, and mechanosensation. Our expression analysis indicated that most iron-related genes are generally upregulated by day 15, with some peaking earlier, suggesting their crucial role in mid-life iron regulation. Lifespan assays revealed that certain mutants of non-transferrin bound iron (NTBI) uptake regulators, such as *smf-1* and *smf-3*, are linked to extended lifespans, while *zipt-17* mutants showed slightly reduced longevity. Mobility assessments indicated significant declines in speed among several mutant strains by day 7, pointing to mobility issues related to altered iron metabolism. Body size measurements varied considerably among mutant strains, with some demonstrating significant changes over time. Behavioral analyses found that most strains exhibited mechanosensory responses similar to wild-type worms at day 1; however, certain mutants displayed different rates of response reduction by day 7. FerroOrange staining confirmed increased iron accumulation with age in most mutant strains, except for *zipt-16* and *zipt-17*, highlighting the connection between iron regulation and aging. Collectively, our current findings demonstrate that iron regulatory genes in *C. elegans* play diverse and critical roles in maintaining iron homeostasis, influencing lifespan, mobility, body size, and behavioral responses throughout the organism's life. These findings deepen our understanding of iron regulation's impact on health and aging in *C. elegans*.

## 1. Introduction

Iron is an essential micronutrient that plays crucial roles in biological processes such as oxygen transport, energy metabolism, and DNA synthesis (Zeidan et al., 2021). As organisms age, the expression of iron regulatory genes can change significantly, potentially affecting iron accumulation and toxicity, which are linked to various age-related disorders (Martins et al., 2022; Ward et al., 2014; Zeidan et al., 2021). However, the mechanisms and patterns by which iron regulatory gene expression changes with age are not well understood. Age-related alterations in iron homeostasis, referred to as age-associated iron dyshomeostasis, can impact multiple functions in humans, including mobility, sensory function, and cognition (Ferreira et al., 2019; Sato et al., 2022; Son et al., 2020; Zeidan et al., 2021). The specific effects of individual iron regulatory genes on mobility and behavior, and how these effects evolve over time, remain largely unexplored. Moreover, while evidence indicates that iron accumulates with age, there is limited information on how different iron homeostasis regulation genes contribute to this age-related tissue iron accumulation.

*Caenorhabditis elegans (C. elegans)* serves as an excellent model for investigating iron homeostasis and aging due to its ease of handling, low cost, and short lifespan. In *C. elegans*, iron homeostasis is regulated by a network of genes that control iron uptake, storage, and export. Notably, *C. elegans* are heme auxotrophs, meaning they cannot synthesize their own heme and must obtain it from their diet, making heme transport genes essential for *C. elegans* (Chen and Hamza, 2023; Rao et al., 2005). Research indicates that disruptions in iron regulation, or more specifically iron accumulation in *C. elegans*, can contribute to age-related protein aggregation, impact longevity, and cause behavioral changes (Hu et al., 2008; Klang et al., 2014; Valentini et al., 2012). This suggests a critical link between iron homeostasis and physiological performance in aging. As many mutant strains related to iron homeostasis are available in *C. elegans*, researchers can study these mutants and gain valuable insights into how both iron deficiency and overload influence the aging process.

In this study, we investigated how aging affects the expression of iron regulatory genes in *C. elegans* and evaluated how disrupting iron homeostasis—via knockout mutants of iron-related genes—impacts lifespan, mobility, body size, and mechanosensory function (nose touch response). Building on prior evidence linking aging to iron accumulation, we also quantified labile iron levels using iron staining at days 1 and 7 of adulthood. By analyzing the expression of iron regulatory genes across different life stages—larval stages, day 1, day 7, and day 15—we aim to gain deeper insights into how aging influences the regulation of iron homeostasis. Using phenotypic analysis of mutants in key iron-regulatory genes, we investigated the broader physiological consequences of disrupted iron homeostasis across multiple domains of organismal function, including mobility (locomotion speed), development (body size), and sensory function (mechanosensation). These measures were chosen because they reflect distinct yet interconnected aspects of organismal health that decline with age and are sensitive to disruptions in iron balance. This integrated approach offers new insights into the intersection of iron metabolism and aging and identifies candidate genes that may underlie sensory decline and age-related functional deterioration.

## 2. Materials and Methods

### 2.1. Strains

Unless otherwise noted, all *C. elegans* strains were maintained at 20 °C on standard Nematode Growth Medium (NGM) plates with OP50 bacteria as the food source. For this study, we identified the primary iron-regulatory genes in *Homo sapiens* and subsequently determined their homologues and orthologues in *C. elegans*. For genes without known orthologues in *C. elegans*, we then conducted a BLAST search to identify potential orthologues. We identified genes involved in various processes of iron homeostasis in *C. elegans*, including: heme iron uptake (*hrg-4(tm2994)*, *mrp-5(ok2067)/szT1*), transferrin receptor for transferrin-bound iron uptake (*gcp-2.1(ok1004)*), non-transferrin-bound iron (NTBI) uptake (*F55H2.5(ok3611)*/DcytB orthologue, *smf-1(eh5)*, *smf-2(gk133)*, and *smf-3(ok1035)*—homologues of DMT1, *zipt-16 (gk251)*, *zipt-17(gk254)*, and *mfn-1*(*tm6321*)), iron export (*fpn-1.1 (tm6914)*, *fpn-1.2(tm14177)*, and iron storage (*ftn-1(ok3625)* and *ftn-2 (ok404))*. All of these mutants are deletion mutants; further details can be found in Supplementary Table 1. All mutant strains as well as the wild type N2 strain were obtained individually from the Caenorhabditis Genetics Center (CGC) or the National BioResource Project (NBRP). The mutants have been validated either by the individual laboratories that generated them or by the CGC or NBRP.

### 2.2. Gene expression quantification- qPCR

To quantify the mRNA levels of iron-related genes, we collected age-synchronized worms at various stages (larval stages L1 and L4, and day 1, day 7, and day 15 adults) for total mRNA extraction. Total RNA was isolated using TRIzol (Invitrogen) according to the manufacturer's protocol. cDNA synthesis was performed using the High-Capacity cDNA Reverse Transcription Kit (ThermoFisher Scientific). All qPCR reactions were conducted in 10 μL volumes, with a primer concentration of 1 μM, mixed with PowerUp SYBR Green Master Mix (ThermoFisher Scientific) and analyzed using a CFX96 Touch Real-Time PCR System (Bio-Rad). The mRNA levels of *act-1* served as a normalization control for all qPCR experiments, and data were analyzed using the ΔΔCt method. We performed 3–4 biological replicates (individual RNA extractions) for each assay, with three technical replicates per biological sample. The qPCR results were plotted using the biological replicates. The primers used in this study are listed in the supplementary Table 2.

### 2.3. Assessment of intracellular iron levels using FerroOrange

To assess intracellular labile iron levels in *C. elegans*, we used the iron-sensitive fluorescent dye FerroOrange (Dojindo Molecular Technologies) to quantify iron accumulation in Day 1 and Day 7 worms. Day 15 animals were excluded from this assay due to their severe immobility and increased fragility, which hindered effective dye uptake and reliable imaging. FerroOrange was prepared at a final concentration of 10 μM in 480 μL of M13 buffer, following the manufacturer's protocol. To promote feeding and dye ingestion, 20 μL of concentrated *E. coli* OP50 bacterial suspension was added to the mixture. Approximately 30–50 worms per strain were washed in M9 buffer to remove residual bacteria and then transferred into the FerroOrange solution. Samples were incubated on a rotator at room temperature for 5 hours to ensure sufficient uptake of the dye. After incubation, the samples were centrifuged to pellet the worms, followed by three thorough washes with M13 buffer to remove excess dye and bacteria.

Meanwhile, a slide was prepared with 100 μL of a thin 2% agarose lawn, which was pre-dried for 5 minutes to create a firm surface for immobilization. Individual worms were carefully transferred onto the slide using a fine eyelash pick. Fluorescence imaging was conducted immediately with an Olympus BX51 fluorescence microscope coupled with a 10X UPlanFI objective and QImaging optiMOS camera. All images were captured with identical exposure settings for consistency across samples and strains. Fluorescence intensity, indicative of intracellular labile iron levels, was quantified using ImageJ software in accordance with image analysis guidelines. Regions of interest (ROIs) were manually drawn around the body of each worm, and the mean fluorescence intensity was recorded. At least 15 worms per strain and age group were analyzed.

Quantification data from each strain were averaged and plotted, and unpaired two-tailed Student's *t*-tests were performed to determine statistical significance between Day 1 and Day 7 values. A *p*-value <0.05 was considered statistically significant. This method offers a reliable approach for evaluating iron levels in *C. elegans*.

### 2.4. Lifespan assay

All lifespan studies were conducted in triplicate at 20 °C, following established protocols (Xiao et al., 2013, 2015; Zhang et al., 2018). For consistency, FUdR (final concentration of 100 μM) was included in all lifespan assays to block reproduction and maintain synchronous populations. The first day of adulthood was designated as day one. Worms were censored if they crawled off the plate, exploded, bagged, or became contaminated, ensuring accurate data interpretation. Worms were counted every other day, and the numbers of alive, dead, and censored worms were recorded. GraphPad Prism was used to generate the lifespan graphs. The comprehensive statistical analysis summaries for all lifespan experiments can be found in Supplementary Table 3.

### 2.5. Assessment of locomotion speed, body length and width

The spontaneous locomotion speed, body length and width of individual worms were evaluated using freshly seeded NGM plates. Each worm was transferred to the plate and allowed to acclimate for ten minutes. For each strain, at least ten worms were tested for one minute at two different ages, Day 1 and Day 7, as severe immobility and fragility in Day 15 worms made reliable assessment technically challenging. The plates were then placed under an automated worm tracking system (Baek et al., 2002), and video was recorded for 45 seconds using a Basler acA2500 camera equipped with a 10X close-focus zoom lens mounted on an MDF MSCOP-010 animal lab IL laminator base (MBF Bioscience). WormLab software 4.1.0 was used to calculate the speed, body length, and body width of the worms. A *t*-test was conducted to assess the significance of differences in speed and size, with *p* < 0.05 considered significant.

### 2.6. Nose touch assay

The nose touch assay was conducted to evaluate mechanosensory responses in *C. elegans*. Worms were placed on plates with a thin layer of bacteria and allowed to acclimate for 30 minutes (Fernandez-Abascal et al., 2022). Forward-moving worms were then prodded with an eyelash hair attached to a picker, and responses were recorded upon reversal or head withdrawal (Fernandez-Abascal et al., 2022). Responses were categorized as follows: no movement, 1 second, 2 seconds, or 3 seconds. If no response occurred or the worm continued moving forward, it was recorded as no movement. For backward movements, the latency was noted according to the timestamps. Each worm was subjected to three touches, and the average response was recorded. For each strain, 10 to 30 worms were tested per trial. Experiments were performed in triplicate on two different dates, assessing worms at two life stages: day 1 and day 7. Results were grouped into two categories: responses detected within 2 seconds of the touch (considered positive) and no response within 2 seconds (considered negative). The percentage of positive responses was calculated, and a *t*-test was conducted to assess the significance of differences in response, with *p* < 0.05 deemed significant.

## 3. Results

### 3.1. The expression levels of iron homeostasis-related genes increase with age

**Fig. 1.**
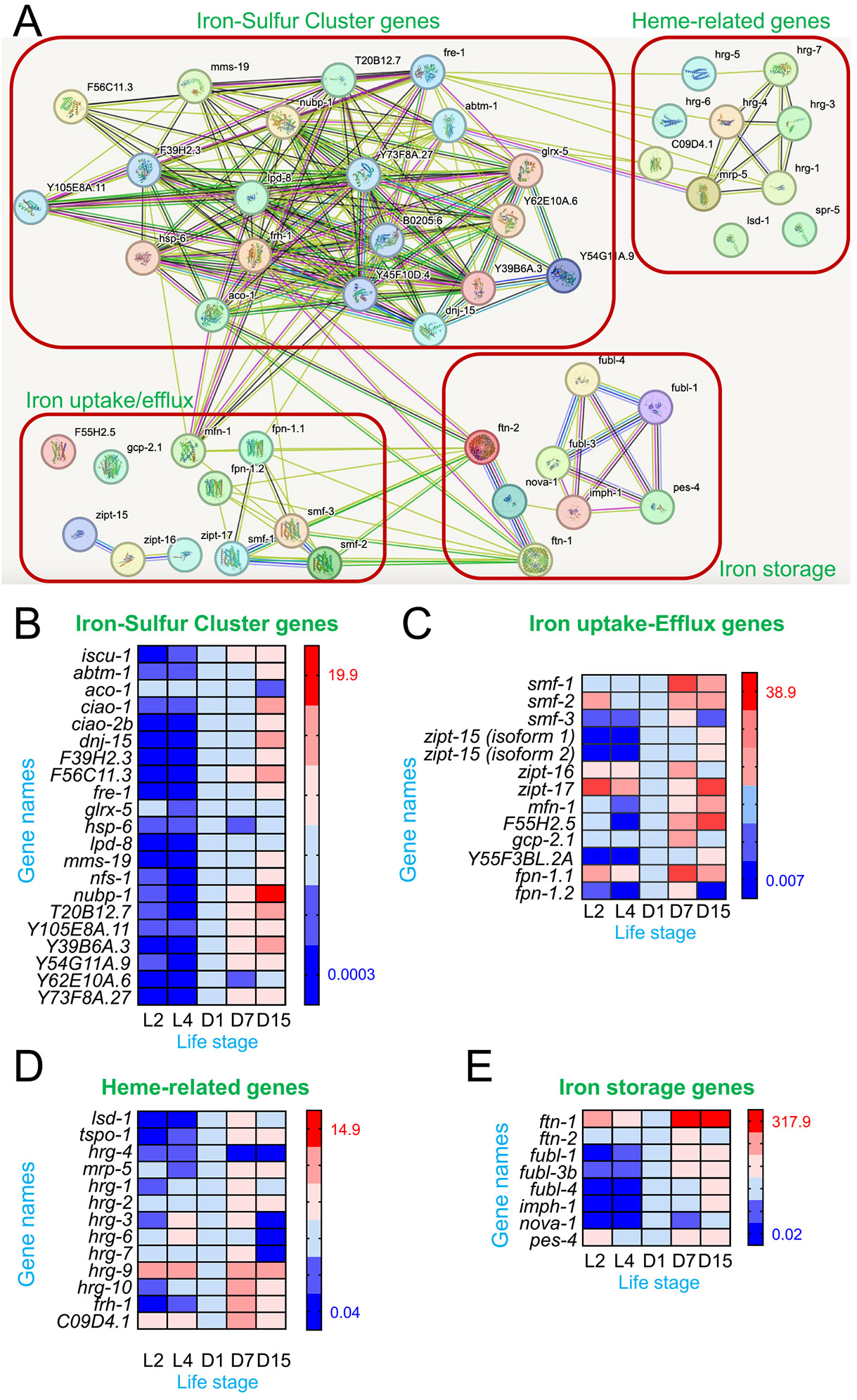
Age-related changes in the gene expression of iron homeostasis regulators in *C. elegans*. (A), String analysis of iron homeostasis-related genes in *C. elegans*. (B-E), Heatmap illustrating changes in gene expression for Iron-Sulfur Cluster genes (B), iron uptake-efflux genes (C), Heme-related genes (D), and iron storage-related genes (E) in *C. elegans* during aging. The data are based on 3 to 4 biological replicates. The color scale indicates the levels of fold change in gene expression. Row-by-row comparisons (e.g., L2 – D15) are provided for each gene, with expression levels normalized to day 1 values for each respective gene.

Similar to many other species, including mice, rats, and zebra fish, *C. elegans* experiences an age-related phenomenon of iron overload (Bloomer et al., 2020; Hamilton et al., 2014; Massie et al., 1983; Xu et al., 2012). As the initial step in systematically studying age-related changes in iron homeostasis, we set to assess the expression levels of iron regulatory genes in *C. elegans* by qPCR across different life stages (Fig. 1). By identifying the *C. elegans* homologues of key iron-regulatory genes in *Homo sapiens* and probing iron-regulatory genes without known homologues, we compiled a list of 59 genes involved in iron homeostasis in *C. elegans*. Through STRING analysis (Szklarczyk et al., 2023), these genes were further grouped into four major categories: iron sulfur cluster genes, iron uptake/efflux genes, heme-related genes, and iron storage-related genes (Fig. 1A).

Next, we analyzed the expression of genes involved in iron-sulfur cluster formation (Fig. 1B), iron uptake and efflux (Fig. 1C), heme transport (Fig. 1D), and iron storage (Fig. 1E) at larval stages (L2 and L4), as well as on days 1, 7, and 15 of adult life. Overall, we found that the iron storage-related genes, especially ferritin *ftn-1* (Fig. 1E), exhibited the strongest age-dependent upregulation among the four major categories of iron homeostasis-related genes. This may represent a compensatory response to mitigate iron overload, consistent with our findings (discussed below) and previous observations in aging *C. elegans* (James et al., 2015; Klang et al., 2014). In addition, our results reveal notable temporal patterns, as illustrated by the colors on the heat maps. Importantly, most genes exhibited increased expression by day 15, indicating a general upregulation of iron-related pathways with age. However, several genes—including *aco-1* (aconitase 1), *smf-3* (divalent metal transporter), *gcp-2.1* (with sequence homology to transferrin receptor), *zipt-16* (zinc and NTBI transporter), *fpn-1.1* and *fpn-1.2* (ferroportin homologues), *lsd-1* (lysosomal sulfatase – iron sulfur cluster), *hrg-1, hrg-3, hrg-7*, and *hrg-10* (heme-responsive genes), *frh-1* (frataxin), *C09D4.1* (uncharacterized protein – likely involved in heme transport), and *ftn-2* (ferritin heavy chain)—peaked earlier at day 7, suggesting a coordinated mid-life shift in iron regulation. Interestingly, around Day 7, *C. elegans* begins to show metabolic changes and subtle declines in physiological function, despite retaining moderate mobility and responsiveness (see results below) (Sinha and Pincus, 2022). These midlife expression peaks may reflect a transitional phase in iron regulation, similar to patterns observed in other models of aging and oxidative stress (Dues et al., 2016). Interestingly, *hrg-4*, a heme transport gene, maintained generally low expression levels but exhibited a significant peak at day 1, suggesting its role in early iron metabolism or heme utilization. This early-life peak suggests a potential role in heme acquisition or utilization during early adulthood, aligning with previous findings that *hrg-4* facilitates dietary heme uptake in *C. elegans*, which is essential for growth and development (Rajagopal et al., 2008). Overall, our data demonstrate that the expression of iron regulatory genes is dynamic and varies considerably across different life stages of *C. elegans*, with distinct subsets of genes peaking at different stages in the later life.

**Fig. 2.**
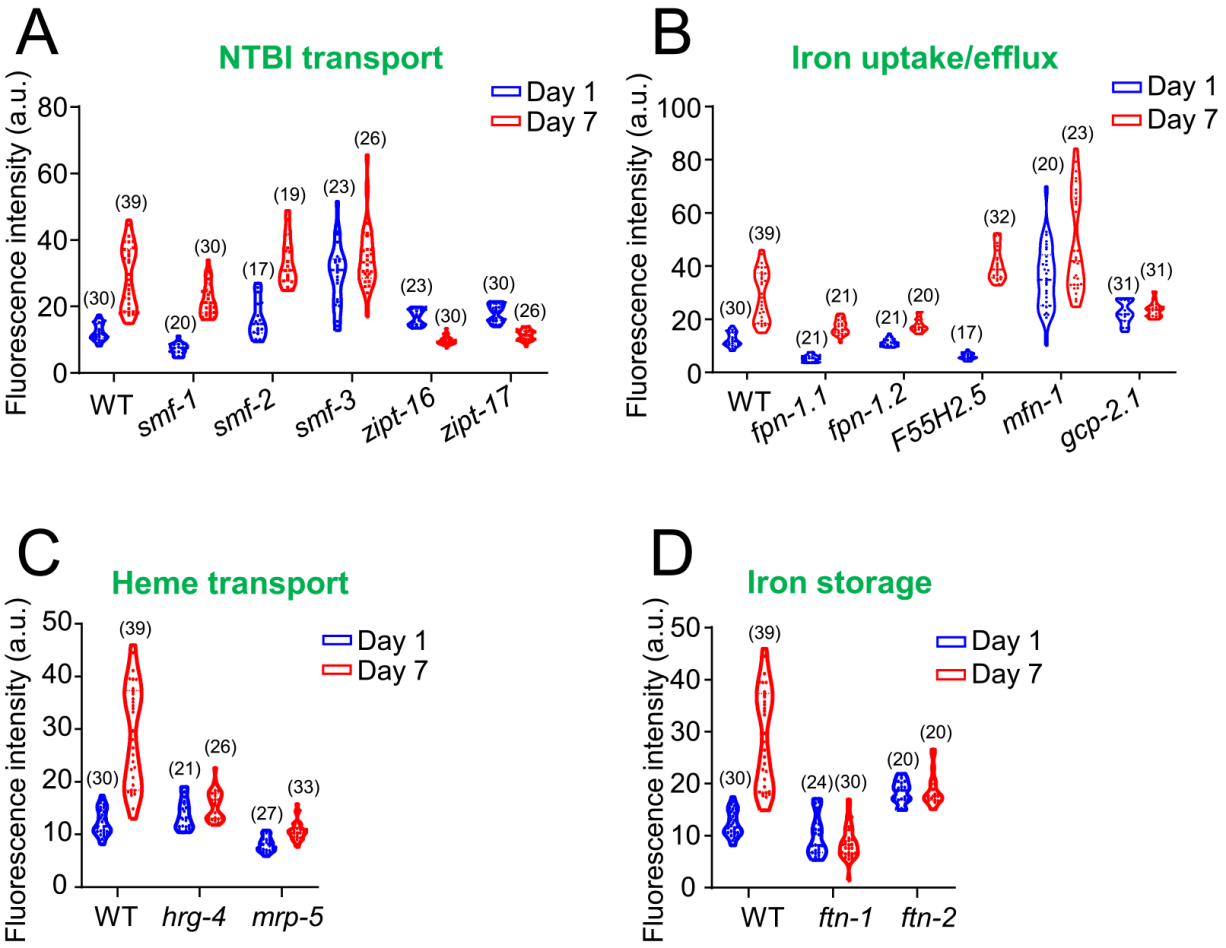
Age-dependent iron accumulation in *C. elegans* assessed by FerroOrange fluorescence quantification of labile iron in wild-type and iron homeostasis mutants. Labile iron levels were measured in Day 1 and Day 7 adult worms using FerroOrange staining in both wild-type and iron homeostasis deletion mutants. Mutants were grouped according to functional categories: non-transferrin-bound iron (NTBI) transport (A), iron uptake-efflux (B), Heme-related (C), and iron storage-related (D). Generally, fluorescence increased by day 7, indicating iron accumulation with age. All mutant strains exhibited significantly different fluorescence levels compared to wild-type worms at each respective life stage. When comparing fluorescence between days 1 and 7 within the same strains, smf-3, ftn-1, and ftn-2 mutants did not exhibit significant changes over this period. *zipt-16* and *zipt-17* mutants displayed reduced fluorescence at day 7 compared to day 1. Violin plots were used to visualize probability distributions, with sample sizes noted in parentheses for each condition. Statistical significance was determined using unpaired t-tests (p < 0.05), and full statistical results are provided in Supplementary Table 4. For this and subsequent figures, the smf and zipt mutant strains are shown together in the “NTBI transport” panel, as they (and their human orthologues) are primarily involved in the uptake of NTBI. In contrast, ferroportin (*fpn-1.1* and *fpn-1.2*), the transferrin homologue *gcp-2.1*, DcytB (*F55H2.5*), and mitoferrin (*mfn-1*), which play distinct roles in cellular iron export, transport, and the movement of iron into and out of the cytoplasm, are represented in a separate “Iron uptake/export” panel, reflecting their specific contributions to iron mobilization.

Notably, for the remainder of this study, most experiments were conducted at two time points rather than three, as shown in the initial gene expression analysis. Days 1, 7, and 15 were chosen to represent early adulthood, midlife, and advanced age in *C. elegans*, capturing key stages of age-related change. Although our initial aim was to assess multiple phenotypes across all three time points, technical limitations made this impractical for certain assays. Specifically, labile iron quantification and behavioral measurements—including locomotion speed, body size (length and width), and nose touch response (Figs. 2 and 4–6)—could not be reliably performed at Day 15 due to the severe immobility and fragility of aged animals. In contrast, gene expression analysis (Fig. 1) remained feasible at this stage, as it required only the collection of worms, independent of their mobility.

### 3.2. The impacts of iron homeostasis regulators on iron levels

After illustrating the age-related changes in the expression of iron homeostasis-related genes, we investigated how disruptions in these processes might impact cellular iron levels. Using a range of deletion mutant strains obtained from two public *C. elegans* consortia (see Methods for details), we employed the small molecular iron-sensing dye FerroOrange for live-cell fluorescence imaging to measure intracellular iron. Of note, while our comprehensive analysis of age-related gene expression changes identified iron-sulfur cluster genes as a major group of iron-related genes (Fig. 1), many lack viable mutants for functional assessment. This is likely due to their essential roles in housekeeping functions, including energy metabolism and DNA replication. As a result, we focused on cellular iron homeostasis regulators to assess their roles in iron overload with age and their impacts on longevity and behavior. Fluorescence data were collected from wild-type (WT) and fourteen iron regulatory mutant worms at days 1 and 7 (Fig. 2).

Notably, many mutant strains exhibited significantly different fluorescence levels compared to WT worms at both time points. Comparisons between days 1 and 7 showed significant increases in fluorescence (suggesting increased iron accumulation) for most mutants, except for the NTBI transporter mutants *zipt-16* (Fig. 2A), *zipt-17* (Fig. 2A), *smf-3* (Fig. 2A), and ferritin mutants *ftn-1* (Fig. 2D), and *ftn-2* (Fig. 2D), which remained stable or even decreased over time. Since most mutants displayed increased fluorescence by day 7, similar to WT worms, these genes may not be essential for age-related iron overload. In contrast, *zipt-16*, *zipt-17*, *smf-3*, *ftn-1*, and *ftn-2* appear to play critical roles in iron accumulation by day 7. *zipt* mutants exhibited reduced iron accumulation at day 7, likely due to disruption of non-transferrin-bound iron transport, which mitigated the age-related buildup of labile iron. Similarly, although heme-responsive gene *hrg-4* mutants showed comparable iron levels to wild-type at day 1, their iron levels remained relatively stable by day 7, indicating a significantly slower rate of iron accumulation with age. This pattern was also observed in the heme transport mutant, *mrp-5*. In contrast, mitoferrin *mfn-1* mutants had notably lower iron levels at day 1, but experienced a dramatic increase in iron accumulation by day 7, surpassing wild-type levels, suggesting a crucial role for mitochondrial iron transport in aging. Finally, *gcp-2.1* mutants exhibited elevated iron levels at both day 1 and day 7 compared to wild-type controls.

### 3.3. The impacts of iron homeostasis regulators on longevity

**Fig. 3.**
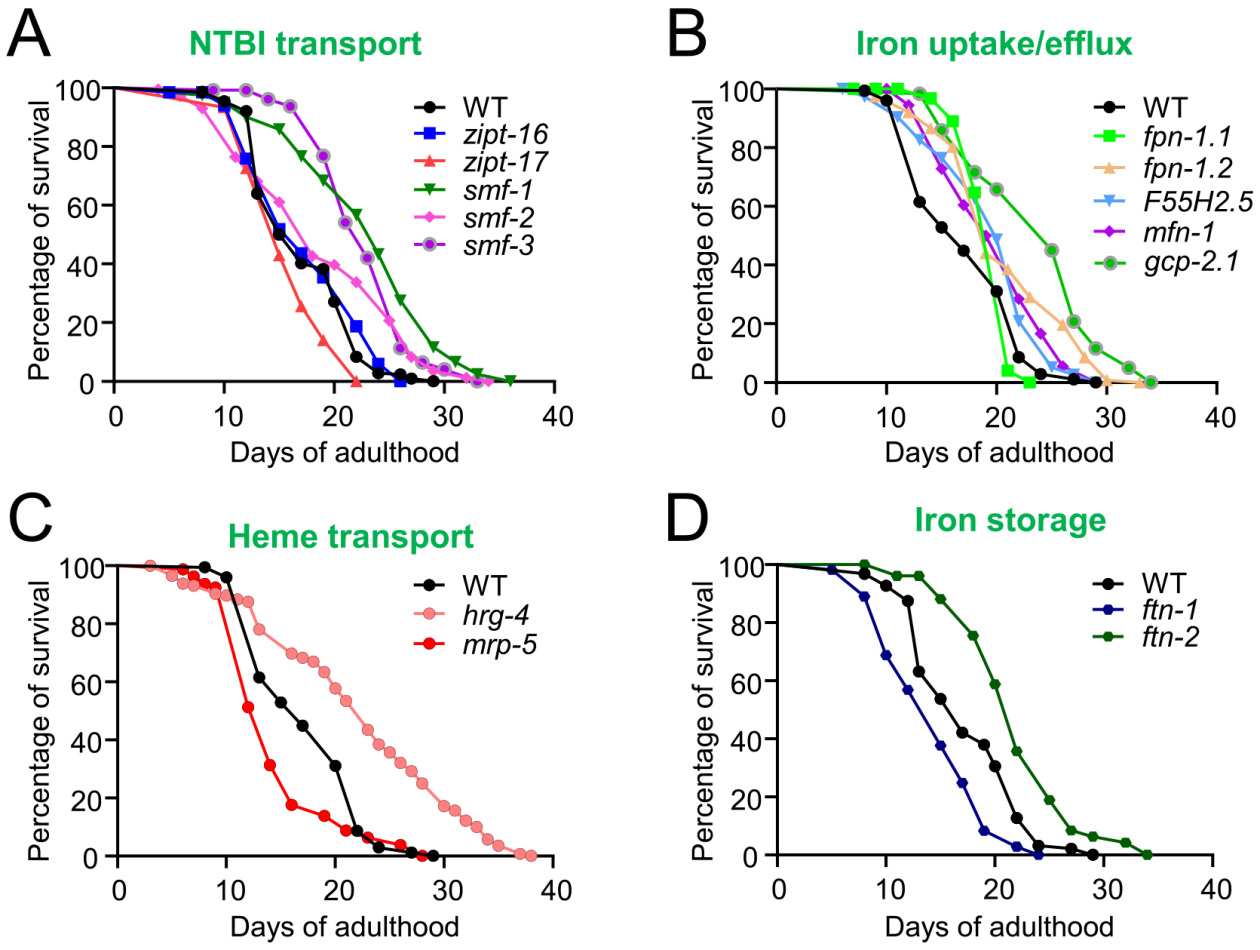
Lifespan analysis of iron regulatory gene mutants in *C. elegans*. (A), Mutations in key non-transferrin-bound iron (NTBI) uptake regulators *smf-1* and *smf-3* lead to extended lifespans compared to wild-type. (B), Ferroportin *fpn-1.2* mutants have an extended lifespan compared to wild-type, whereas *fpn-1.1* mutants display a shorter lifespan. (C), Heme transporter *mrp-5* mutants have a shorter lifespan compared to wild-type, while *hrg-4* mutants demonstrate an extended lifespan. (D), Iron storage protein *ftn-1* mutants exhibit a shorter lifespan, whereas *ftn-2* mutants show a longer lifespan.

Given that many genes involved in iron homeostasis exhibit age-dependent expression changes, we next investigated the impact of key iron regulatory genes on longevity using lifespan assays (Fig. 3). For NTBI uptake regulators (Fig. 3A), both *smf-1* and *smf-3* mutants demonstrated significantly longer lifespans than WT individuals, suggesting that reduced NTBI uptake may promote longevity. We also examined two predicted zinc ion transporters (implicated in NTBI uptake) that can often transport both zinc and iron (Earley et al., 2021), *zipt-17* and *zipt-16*. The *zipt-17* mutant showed a slightly shorter lifespan, while the *zipt-16* mutant displayed no significant lifespan changes, indicating that these transporters may not be critical for iron uptake.

In the analysis of putative genes involved in the iron transport, both *fpn-1.2* and *gcp-2.1* mutants led to significant lifespan extension (Fig. 3B), while the ferroportin *fpn-1.1*, mitoferrin *mfn-1*, and *F55H2.5* (human CYB561 ortholog) mutants had modest effects on lifespan, suggesting that these genes may not significantly impact overall longevity in *C. elegans* under the conditions tested. Regarding heme transport genes (Fig. 3C), the *mrp-5* mutant led to a reduced lifespan, whereas the *hrg-4* mutation significantly extended lifespan, underscoring their distinct roles in heme utilization and aging. Lastly, the analysis of iron storage genes (Fig. 3D) revealed contrasting results: the *ftn-1* mutant shortened lifespan, while the *ftn-2* mutant extended it. Although both genes encode ferritin subunits, previous studies suggest that they are expressed in different tissues, may respond differently to elevated iron levels, and likely serve distinct roles in iron sequestration during aging (Anderson and Leibold, 2014; Gourley et al., 2003; Kim et al., 2004). The contrasting lifespan outcomes observed in *ftn-1* and *ftn-2* mutants highlight the need for further investigation into their distinct expression patterns and interactions with iron-sensing and stress-response pathways. These findings highlight the differential effects of iron storage genes on organismal health and longevity, likely through their distinct roles in managing iron toxicity and oxidative stress.

### 3.4. The impacts of iron homeostasis regulators on mobility

**Fig. 4.**
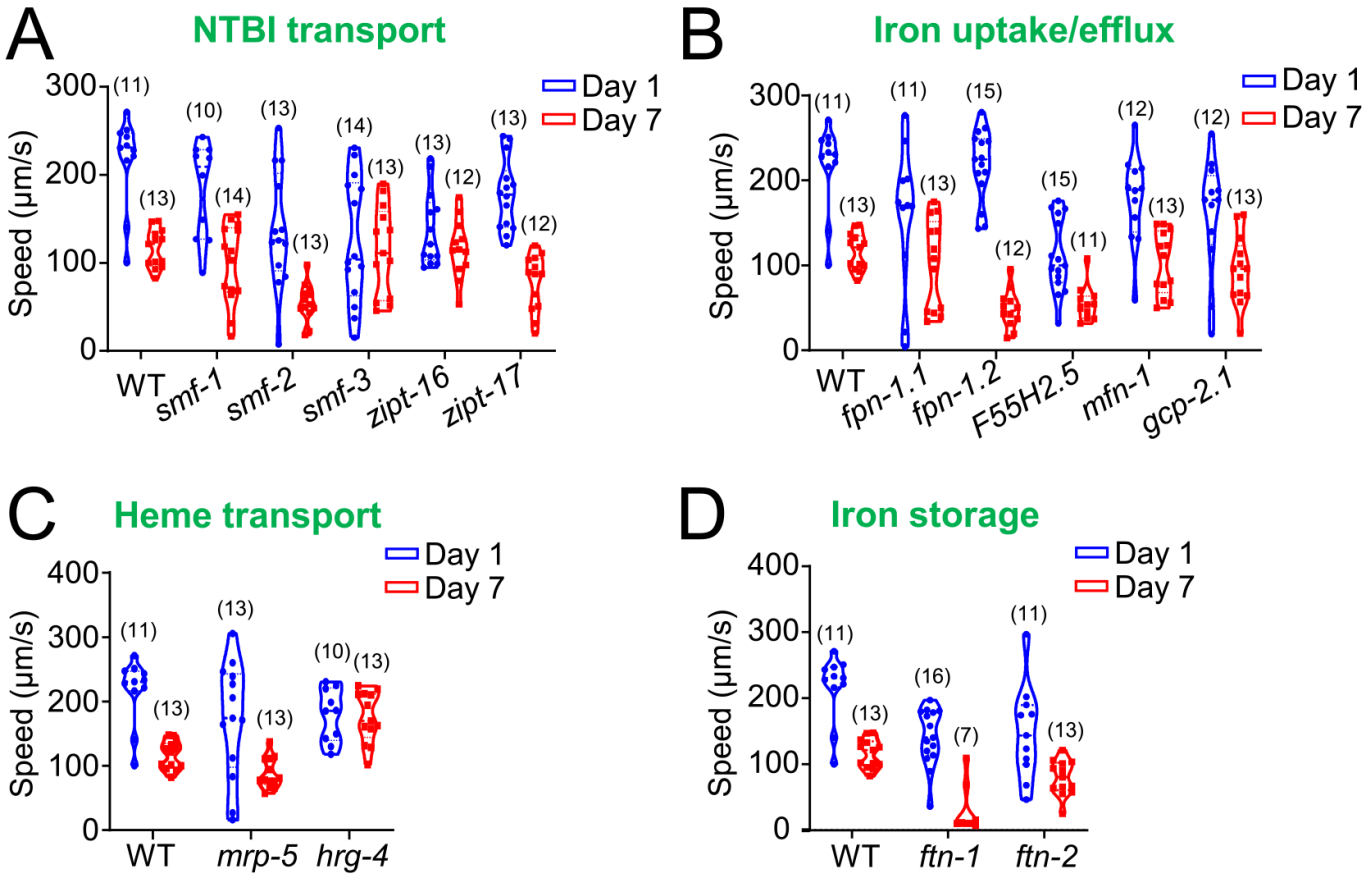
Locomotion speed analysis of *C. elegans* iron regulatory gene mutants at days 1 and 7. (A), Mutations in key NTBI uptake regulator cause a significant reduction in locomotion speed by day 7, except for *smf-3* and *zipt-16* mutants. At day 1, significant speed differences are observed between WT and most NTBI regulatory gene mutants, with the exception of *smf-1*. By day 7, most NTBI mutants show significant differences in speed compared to WT, apart from *zipt-16*. (B), Most iron uptake/efflux regulator mutants exhibit no significant speed differences at either life stage, except for *fpn-1.2* mutants at day 7 and *F55H2.5* mutants, which show significant reductions in speed at both days. (C), Heme transport mutants show no significant speed differences compared to WT at day 1 but exhibit significant reductions by day 7. (D), Both *ftn-1* and *ftn-2* iron storage mutants display significantly lower speeds compared to wild-type at both day 1 and day 7. Probability distributions were represented as violin plots, with sample sizes provided in parentheses for each condition. An unpaired t-test was used to assess statistical significance, defined as p < 0.05, and full statistical results are provided in Supplementary Table 4.

Age-related mobility decline is commonly observed across many species, including *C. elegans* (Altun et al., 2007; Bair et al., 2019; Ballak et al., 2014; Mondino et al., 2023). Additionally, locomotion speed reflects physical function, neuromuscular integrity and general physiological robustness, which typically decline with age and are widely used as proxies for healthspan in *C. elegans* (Hahm et al., 2015; Huang et al., 2004). For that, using Wormlab, we assessed the speed of various iron regulatory mutants at day 1 and day 7 (Fig. 4A). Our findings indicate that while some NTBI transporter mutants showed a significant decline in speed by Day 7 (including *smf-1*, *smf-2*, and *zipt-17* mutants), others maintained normal locomotion when comparing Day 1 to Day 7 (including *smf-3* and *zipt-16)*. For the putative iron efflux regulators, no significant speed differences were observed at either time point, except for the *fpn-1.2* mutant at day 7 and *F55H2.5* mutant at both time points, which exhibited reduced speed (Fig. 4B). In addition, while heme transport regulators showed no significant speed differences at day 1, the *mrp-5* mutant demonstrated reduced mobility by day 7 whereas the long-lived *hrg-4* mutant exhibited increased speed compared to WT at that time (Fig. 4C). Lastly, iron storage gene knockouts consistently displayed significant speed reductions compared to WT at both day 1 and day 7 (Fig. 4D). Collectively, our results show that specific iron-related mutants in *C. elegans*, including *smf-1*, *smf-2*, *zipt-17*, *fpn-1.2*, *F55H2.5*, *mrp-5*, and iron storage genes, experience reduced mobility with age. In contrast, the long-lived *hrg-4* mutant maintains faster movement by day 7, underscoring the differential roles of iron regulation pathways in locomotion.

### 3.5. The impacts of iron homeostasis regulators on body size

Body size in *C. elegans* is a sensitive indicator of physiological status, reflecting changes in tissue integrity, metabolic balance, and overall health across the lifespan. While body size typically increases in length and width during early adulthood as part of normal maturation, age-related changes can signal underlying physiological alterations. These changes in body size often precede or coincide with other signs of decline, making it a valuable proxy for assessing healthspan. Furthermore, altered lifespans frequently correspond with changes in developmental growth and body size compared to wild-type, underscoring its relevance to longevity-related mechanisms (Gems et al., 1998; Kenyon et al., 1993; Lee et al., 2016; Y. Zhang et al., 2009). Thus, monitoring body size provides key insights into how disruptions in iron metabolism may contribute to age-related functional decline and impact longevity.

**Fig. 5.**
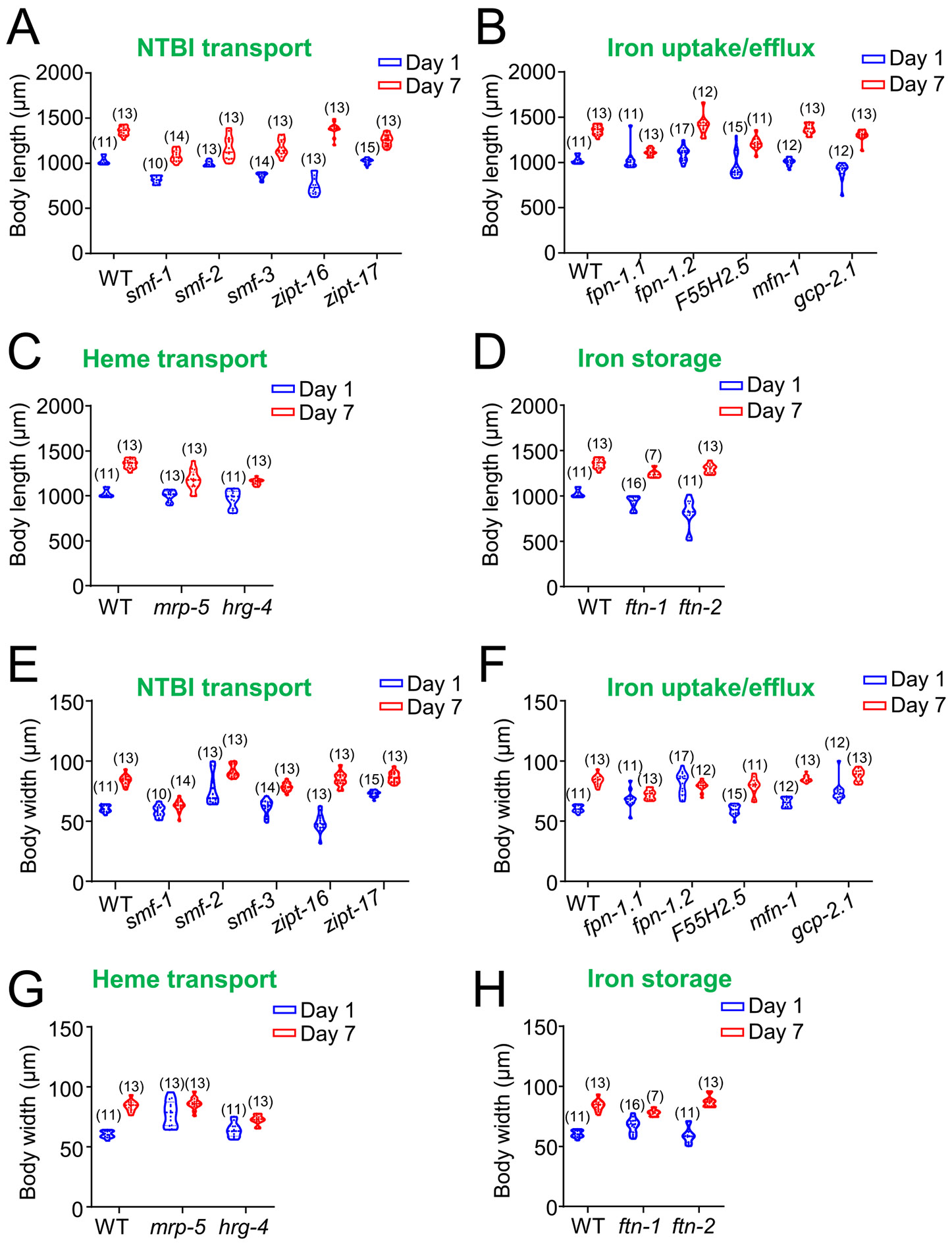
The impacts of iron-related genes on the *C. elegans* body sizes. (A), Mutants of major NTBI uptake regulators show significant differences in length compared to WT controls at both life stages, except for *smf-2* and *zipt-17* (shorter) at day 1, and *zipt-16* at day 7. (B), Mutants of iron uptake/efflux regulators show no significant length differences, except for *fpn-1.1*, *F55H2.5*, and *gcp-2.1* (shorter) at day 7, and *fpn-1.2* (increased) and *gcp-2.1* (shorter) at day 1. (C), Heme transport mutants show no significant length differences at day 1, but by day 7, all heme transport mutants exhibit shorter length compared to WT. (D), Mutant of iron storage genes exhibit no significant length differences at either time point compared to WT. (E), Mutants of major NTBI uptake regulators show significant differences in width at day 1, except for *smf-1* and *smf-3*. *smf-2* and *zipt-17* mutants have increased width at day 1, while *zipt-16* mutants show reduced width. By day 7, *zipt-16* and *zipt-17* mutants show no difference in width, but *smf-1* and *smf-3* mutants display reduced width, while *smf-2* knockouts maintain larger width compared to WT. (F), Mutants of iron uptake/efflux regulators show no significant width differences at day 1, except for *fpn-1.2*, *mfn-1*, and *gcp-2.1* mutants, which show increased width. At day 7, only *fpn-1.1* and *fpn-1.2* show significant differences, with reduced width compared to WT. (G), Mutants of heme transport regulators, such as *mrp-5*, show no significant width differences at day 7 but increased width at day 1 compared to WT. *hrg-4* mutants exhibit significantly smaller width at day 7 but no significant differences at day 1. (H), For iron storage genes, *ftn-2* mutants show no width differences compared to WT, while *ftn-1* mutants display increased width at day 1 and decreased width at day 7.

In our analysis of iron homeostasis mutant strains, we noted differences in body length at both day 1 and day 7. Several NTBI uptake mutants, such as *smf-1* and *smf-3*, exhibited significant reductions in body length compared to WT at both time points (Fig. 5A). While most iron uptake/efflux mutants showed no significant length differences from WT, the *fpn-1.2* mutant was significantly longer than WT at day 1, though this difference disappeared by day 7 (Fig. 5B). Heme transport mutants showed significant reductions in length by day 7 (Fig. 5C), while iron storage mutants displayed no significant length differences from WT at any stage (Fig. 5D).

Regarding body width, we observed significant differences in some iron homeostasis mutant strains at both day 1 and day 7 compared to WT controls. Major NTBI uptake regulators had distinct effects on width. At day 1, *smf-2* and *zipt-17* mutants exhibited significantly increased width, while the *zipt-16* mutant displayed reduced width compared to WT. By day 7, *smf-1* and *smf-3* mutants had decreased width, whereas the *smf-2* mutant maintained a larger width (Fig. 5E). Most iron uptake/efflux mutants showed no significant differences at day 1, except for *fpn-1.2*, *mfn-1*, and *gcp-2.1*, which were wider. At day 7, *fpn-1.1* and *fpn-1.2* mutants exhibited smaller widths compared to WT (Fig. 5F). Among heme transport regulators, the *mrp-5* mutant exhibited increased width at day 1, while the *hrg-4* mutant showed significantly reduced width at day 7 (Fig. 5G). In the iron storage regulators, the *ftn-1* mutant showed increased width at day 1 but decreased width at day 7, while the *ftn-2* mutant did not differ significantly at either time point (Fig. 5H).

### 3.6. The impacts of iron homeostasis regulators on mechanosensation

**Fig. 6.**
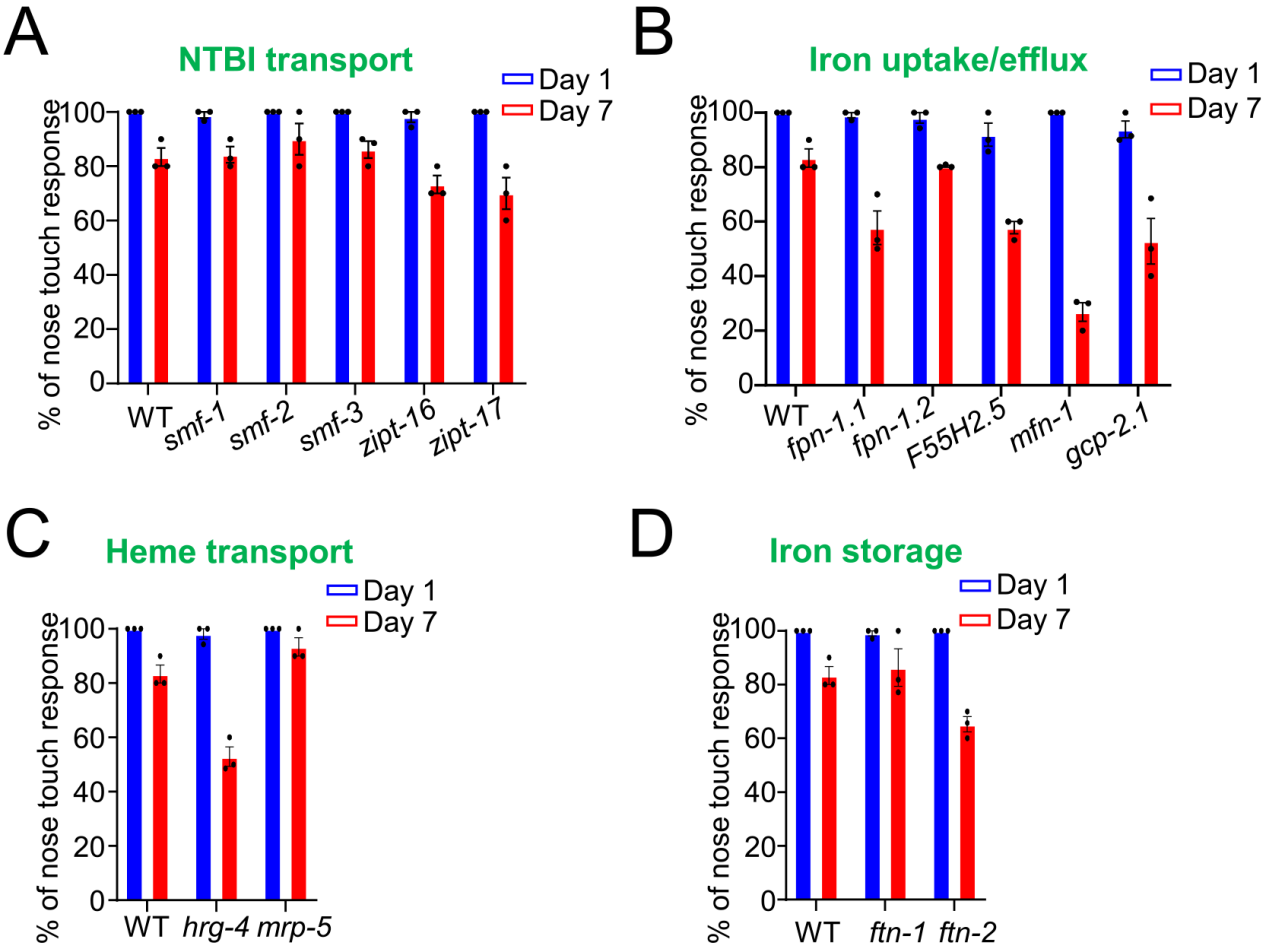
Nose touch responses of *C. elegans* iron regulatory mutants at day 1 and day 7. The nose touch responses of various mutant strains involved in iron regulation were assessed at different life stages, with comparisons made across key categories: major NTBI uptake regulators (A), iron uptake/efflux genes (B), heme transport genes (C), and iron storage genes (D). At day 1, there were generally no significant differences between the mutants and WT worms. However, by day 7, several mutants, including *ftn-2*, *fpn-1.1*, *mfn-1*, *F55H2.5*, and *hrg-4*, showed significantly reduced responses compared to WT. When comparing responses from day 1 to day 7 for each mutant, most showed reduced responses by day 7, except for *mrp-5*, which exhibited no significant changes. These experiments were performed in triplicate, with 30 worms of each mutant assayed in the first replicate, and 10 worms per mutant in the second and third replicates. Violin plots depict the probability distributions, with sample sizes shown in parentheses for each condition. Statistical significance was evaluated using unpaired *t*-tests (p < 0.05), and detailed statistical results are available in Supplementary Table 4.

Mechanosensation (nose touch response) evaluates sensory neuron function and reflexive behavior, both of which decline with age and serve as indicators of functional deterioration (Chalfie et al., 2018; Chew et al., 2013; Pan et al., 2011). On day 1, all iron regulatory mutants demonstrated comparable nose touch responses to those of WT worms, indicating no significant differences in mechanosensation at this early time point. By day 7, while the majority of mutants continued to exhibit similar responses as the WT, *fpn-1.1* (Fig. 6B), *mfn-1* (Fig. 6B), *F55H2.5* (Fig. 6B), *hrg-4* (Fig. 6C), and *ftn-2* (Fig. 6D) mutants displayed significantly reduced responses, suggesting a potential impairment in their sensory function linked to iron regulation and age-related decline. When comparing responses from day 1 to day 7, most mutants showed decreased responsiveness at day 7, except for *ftn-2*, *zipt-17*, and *mrp-5*, which showed no significant changes, indicating preserved mechanosensation with age in these mutants.

## 4. Discussion

Iron is a vital element for nearly all living organisms, yet its overload is linked to numerous adverse conditions in humans (Zeidan et al., 2021). While the link between iron metabolism and aging is well established, with aging often associated with increased cellular iron accumulation (Zeidan et al., 2021), systematic analyses of iron homeostasis-related genes remain limited. Our current study of iron regulatory genes in *C. elegans* reveals dynamic changes in gene expression and physiological responses across different life stages, providing valuable insights into the interplay between iron homeostasis, aging, and overall health. Our gene expression data demonstrate a significant upregulation of iron-related pathways by day 15, underscoring the growing importance of iron regulation as the organism ages. This increase contrasts with certain genes that peaked earlier at day 7, suggesting that they may play key roles in regulating iron homeostasis during early adulthood. Notably, the heme-transport related gene *hrg-4* exhibited low expression levels but showed a pronounced peak at day 1, suggesting its potential role in early heme transport. To our knowledge, this study is the first comprehensive assessment of iron regulatory gene expression in *C. elegans* from the larval stage through youth and into older adulthood.

Building on our understanding of how age affects the expression of iron regulatory genes, we next investigated the impact of individual iron homeostasis genes on longevity. Utilizing various mutant strains, we conducted a series of assays to elucidate the functional roles of these specific iron regulatory genes in lifespan regulation. Interestingly, mutations of several NTBI uptake regulators, such as *smf-1* and *smf-3* (the human DMT-1 orthologs), were associated with extended lifespans. This suggests that limiting iron accumulation, especially labile iron, may alleviate age-related toxicity. These findings are consistent with the hypothesis that limiting intracellular accumulation of labile iron—a redox-active form that can catalyze reactive oxygen species (ROS) via Fenton chemistry—may offer protective effects and potentially delay aging (Klang et al., 2014; Schiavi et al., 2023). In contrast, the *zipt-17* mutant has a significantly shorter lifespan, corroborating previous findings and highlighting how alterations in iron uptake mechanisms can markedly influence longevity. This corroborates previous findings that implicate ZIP transporter family members in maintaining metal homeostasis (iron and zinc balance) under stress conditions, which is essential for organismal viability and can significantly impact lifespan (Aydemir et al., 2012; Novakovic et al., 2020). Furthermore, *ftn-1* mutants showed shortened lifespans, consistent with previous studies highlighting this gene's role in longevity (Kim et al., 2004; Valentini et al., 2012). This finding underscores the importance of proper iron storage and the regulation of labile iron levels in promoting healthy aging. Lastly, the effects of heme transport genes were also distinct. Two characterized heme transport (*mrp-5* and *hrg-4*) mutants were assayed (Korolnek et al., 2014; Sinclair and Hamza, 2015; Yuan et al., 2012). While the *mrp-5* mutation resulted in decreased lifespan, the *hrg-4* mutant exhibited extended longevity, emphasizing the nuanced roles these genes play in heme utilization and regulation.

Our assessments of mobility and body size among various iron signaling-related mutants revealed that the disruption of specific iron homeostasis genes had a significant impact on these physiological traits. Notably, the marked reductions in speed observed in many NTBI uptake mutants by day 7 suggest a potential decline in overall health and motility linked to altered iron metabolism. This aligns with previous observations in mammals, which suggest that reduced motility and physical function are linked to increased iron accumulation (DeRuisseau et al., 2013; Seo et al., 2008; Xu et al., 2008). Additionally, variations in length and width among multiple mutants highlight how iron regulatory genes influence physical development, with some strains showing notable increases or decreases in body dimensions over time. Collectively, these findings provide novel insights into the effects of distinct iron regulatory genes on mobility in aging worms.

We employed mechanosensation as a straightforward assay to assess the sensory functional consequences arising from disruptions in iron regulatory genes. At day 1, most mutant strains exhibited responses to nose touch stimuli that were comparable to those of WT worms. However, by day 7, we observed a notable divergence in the mechanosensory responses of certain mutant strains, with a subset showing significant reductions in their ability to respond to touch. This decline in mechanosensation in specific mutants suggests an age-related deterioration of sensory function, which may be intricately linked to the regulation of iron within the organism. The fact that only certain strains exhibited this decline implies that the affected iron regulatory genes play crucial roles in maintaining sensory function as the animal ages. These findings highlight the potential impact of iron dysregulation on neural health and sensory processing, suggesting that age-associated disruptions in iron homeostasis could contribute to broader age-related sensory impairments. Overall, these findings are consistent with studies in other species showing that age-related iron dysregulation contributes to neurodegeneration and cognitive decline (Biel et al., 2021; D'Mello and Kindy, 2020; Hare et al., 2013), suggesting that it may also impair sensory function, thus warranting further investigation. This connection underscores the importance of iron regulation not only in metabolic processes but also in preserving the integrity of sensory functions throughout the lifespan of the organism.

## 5. Conclusion

In summary, our current findings demonstrate that iron regulatory genes in *C. elegans* play diverse and critical roles in maintaining iron homeostasis, influencing lifespan, mobility, body size, and behavioral responses throughout the organism's life. We propose that the distinct phenotypes observed across mutants reflect a nonlinear relationship between iron levels and organismal health, where both iron deficiency and overload can be harmful, depending on cellular context, timing, and pathway compensation—consistent with hormesis models of aging (Andrews, 2008). A deeper understanding of these relationships may shed light on the consequences of iron dysregulation in aging and age-related diseases in more complex organisms. These results highlight the need for further research to elucidate the mechanisms driving these observed effects.

Building on our findings, further mechanistic studies are needed to clarify how iron metabolism influences lifespan, healthspan, and functional decline in *C. elegans*. These include tissue-specific knockdowns and reporter analyses to delineate the roles of iron-regulatory genes in key tissues such as the intestine, muscle, and neurons, as well as assays to assess their impacts on mitochondrial function, oxidative stress, proteostasis, and ferroptosis.

## Supplementary Material

1

## Data Availability

The datasets are available from the corresponding author on reasonable request. Violin plots represent probability distributions, with sample sizes indicated in parentheses for each condition. Statistical significance was assessed using an unpaired t-test, with p < 0.05 considered significant, and full statistical results are provided in Supplementary Table 4.

## Appendix A. Supplementary data

Supplementary data to this article can be found online at https://doi.org/10.1016/j.exger.2025.112826.
