# Supplementary material for: Exploring age-related iron dysregulation: effects on longevity, body size, and behavior in C. elegans
Source: Exp Gerontol. Author manuscript; Available in PMC 2026 May 12. (PMC13162325; doi:10.1016/j.exger.2025.112826)
Supplement: 1 [file NIHMS2169374-supplement-1.docx]

**Supplementary data:**

**Table 1: List of *C. elegans* iron-related deletion mutant strains used in the study.**

| **Gene Name** | **Mutant Allele** | **Source** |
| --- | --- | --- |
| *hrg-4* | tm2994 | NBRP |
| *mrp-5* | ok2067/szT1 | CGC |
| *gcp-2.1* | ok1004 | CGC |
| *F55H2.5* | ok3611 | CGC |
| *smf-1* | eh5 | CGC |
| *smf-2* | gk133 | CGC |
| *smf-3* | ok1035 | CGC |
| *zipt-16* | gk251 | CGC |
| *zipt-17* | gk254 | CGC |
| *mfn-1* | tm6321 | NBRP |
| *fpn-1.1* | tm6914 | NBRP |
| *fpn-1.2* | tm14177 | NBRP |
| *ftn-1* | ok3625 | CGC |
| *ftn-2* | ok404 | CGC |

**Table 2: Primer list for this study, organized by iron-related gene function.**

| **Gene name** | **Forward (5' to 3')** | **Reverse (5' to 3')** |
| --- | --- | --- |
| *abtm-1* | GACTTCATCGTTGGCTCTGCTC | GCGATTCGTGTTCCTGCCAT |
| *aco-1* | CTGTCACAATTACCAACCACGGA | GTGATGGCAGCAATAACTACGGA |
| *ciao-1* | TCTACAGCCACACACACAGGA | GTAGAATCTGATTGATGAGTCGTAGC |
| *ciao-2b* | CGACCTGTTACTGGCACTGAA | TGGATGCTCTGGATCGTTGA |
| *dnj-15* | ACAACCAGTCACAAGTGGACAA | TTGTAGCTGGTGAAATTTCTGCTT |
| *F39H2.3* | GATCTCAAGAATTGGCGGAGT | TTCCTGTCCAACATAACATCCTT |
| *F56C11.3* | ATGCGTCAGTGTAGAGGATATGATG | ACTTCGCTCCTGTGCTCGTG |
| *fre-1* | GCACTCGGCCTAACAGAAGA | GTGAGAAGTGTCGTTTTTGCTGA |
| *glrx-5* | GAGCCAGCGTGTGGATTCTC | GCTCCTGATCGGTCAGAACG |
| *hsp-6* | GTGTCATCAACGAGCCAACTG | CACCTCCAAGATCGTAGACAGC |
| *iscu-1* | ACTTCAGTTGCTCAGTATCACGAGA | GATTCCTGTTCCGACGCTTG |
| *lpd-8* | AATGCTCACAAGACTGAACCG | GTGTCTCCTGAACTTGAATATACATTG |
| *mms-19* | TTTCGCGATTTGCAACAGGG | TCCGCGATATGGTGTTGTTCA |
| *nubp-1* | AAGATTCAGGATAGGTTCAGCAGA | GAGCAAGATTCGATGTGAGAGTACT |
| *T20B12.7* | CTGAACCTAAGAGCAGTTGTGGA | TGAATGGTGGTTGTCCGAGA |
| *Y105E8A.11* | GGAATTGATGGATGTACTCGTGT | ACCACGTCGAACATTGATCC |
| *Y39B6A.3* | AATTCGGTGGAGCAACTGCA | GGCTTCGTTGGTAAGTGTGAGAG |
| *Y54G11A.9* | AAGTGACGAATAAGGCAGCGA | CACCTCCATCCACCTCAAGTC |
| *Y62E10A.6* | ATTAGTTCGGTATGGTGTGGCT | AATGTAATGTCTCGTCCGATGTTCA |
| *Y73F8A.27* | TATGACGTCAAGTGTGCGGAA | GATATTCACCACCTCGTCTTCACT |
| *smf-1* | AAGACTTGGAGTTGTCAGTGGAA | CCAGAGAATGATGCGAGGAA |
| *smf-2* | TTGCTTGCGAACTTATGAACG | GAGCATCCAGTTCCAGTGTTCT |
| *smf-3* | CATCGGAACTGCCATCTCGT | GAACAAGAAGGTGAAGGTATCGC |
| *zipt-15 (isoform 1)* | ATCACACTATTCGTCGGTCTCG | CCATTCTCATCTACTTCGTCCAT |
| *zipt-15 (isoform 2)* | TTCAATTCTCGGCGTCTACG | GGCGATGGTGAATGTGTTGA |
| *zipt-16* | TACACAGAAGCCGTCAACCTG | GCACGGTAATAGAAGTATTCCAAGT |
| *zipt-17* | TGTTATTGTTCTCTCCATCTACATTCTC | ACTGAAGATGTAATAACTGGCGACT |
| *mfn-1* | CGGATTCTCGTGTGGATTACAAG | AACGGACCAGGAGAGAGCAG |
| *F21D5.3A.1* | TTGCTCATTGTCACTTGGAACT | GAAGACACCACAATCATGCG |
| *F55H2.5* | TTGTCTCGATTACTGTAGCCATGG | CCGACGAATGAAGATGTTGC |
| *gcp-2.1* | CATGGAGATGGAGATGCACTC | GAACTGGTATTGAAGGTAGAACGC |
| *Y55F3BL.2A* | ATCACACTATTCGTCGGTCTGG | CCATTCTCATCTACTTCGTCCAT |
| *fpn-1.1* | TTCATGTTCAACGTGTTCTATCATG | ACTGGCTGTCTTCCGAGGTG |
| *fpn-1.2* | CAACAACCGAATAATCGACGAA | AGAGACACAGAATGACCAAGCC |
| *fpn-1.3* | CTAGTGGTCCAAGCGTTCTCA | CATGTCCAGGCACCAGAGAC |
| *lsd-1* | GGCCAGAGTCAATTAACCAGTT | TCCGACATAGTGAGTAGTGAGAGTTC |
| *tspo-1* | TGGAAGAATACCACTGTTGTCAGT | AAGCGGTCCATACAGCGAAT |
| *F22B5.4* | AATATTCTCGTAATTGGTCAAGAGATC | AGAGTGGATATACTTCATAAGCGTGA |
| *mrp-5* | ATACCTCGTGGCTCTTGTCG | GTGTCGCAGCAGTGAGAGAA |
| *hrg-1* | GAGGAACAACAAAGACAATCGTGC | ATTGCGAACACAGTTCCAGC |
| *hrg-2* | CCAACAGTTAATGCGACGCCAAA | TGACAAGTTTGGTACGGTATCGAT |
| *hrg-3* | GGTCAATTTCACAAGGTCTTG | CCAAAAACCTTCTTCTGAATCAG |
| *hrg-4* | GTGCAGGATCTATCCAAGCAATG | TCGGAAGCTGTTCCAATTTTGTT |
| *hrg-6* | GCTGGTCTTTGTGTTGTCACAT | GCAACCCAGTTCTTCCAAGC |
| *hrg-7* | GTCCAACTTCCCAAATCAATGG | TGACCCAGATGATACTCCTCC |
| *hrg-9* | GTCAACTGAAGGATGAGTACCA | TGTAGGTGGACTATTTGAGACG |
| *hrg-10* | GTGGTACTTGGCTAGGAATGG | TTGACAATTCCACCTCTGCTAG |
| *frh-1* | CGATTCATTTCCGGTTTCCGAAC | AGACATCGGACTGGACAACC |
| *C09D4.1* | CGACGCATCCAGACTCTTTCT | TCGAGGTGGAATGCCTAATGTG |
| *ftn-1* | TATCACGATGAAGTCGAAGCG | AGATAGACATAGGAGGCGTAGAGTTC |
| *ftn-2* | ATCAATAATGTCTCTCGCTCGTCA | AGGTAGACGTATGAGGCATAGAGC |
| *fubl-1* | TTTCTCACTCGCAATCCGCT | TGTGAGTAGCTTGCTGCGG |
| *fubl-3b* | GCGTCTCCTCTTCGACACAT | GGCACAGCTTCGTATTCGTC |
| *fubl-4* | TCTGAGAATGCGAACACTCCAAT | TCCTCCACGACCAATAACCAA |
| *imph-1* | GGAATACTTCAATGCGGGAGGAG | GCTGATATGTTTCGTAGTCGGTTGC |
| *nova-1* | AACAAGCGCAATGGAAGAAATAATG | TCTGAAGCGTCTTGAGTCCG |
| *pes-4* | GAGCATACGCAGCACAGACAA | CAGTAGGTGGTCCGATGAGGT |
| *act-1* | GCTGGACGTGATCTTACTGATTACC | GTAGCAGAGCTTCTCCTTGATGTC |

**Table 3: Statistics of lifespan studies.**

| **Strain** | **Mean Lifespan ±SEM** | **Median Lifespan** | **75% Lifespan** | **n (assayed/total)** | ***P* value against control** | **Fig.** |
| --- | --- | --- | --- | --- | --- | --- |
|  |  |  |  |  |  |  |
| WT | 17.077±0.308 | 17 | 22 | 214/216 | - | 3A |
| *zipt-16* | 17.552±0.436 | 17 | 22 | 133/136 | 0.211 | 3A |
|  |  |  |  |  |  |  |
| WT | 17.077±0.308 | 17 | 22 | 214/216 | - | 3A |
| *zipt-17* | 15.483±0.319 | 15 | 19 | 121/122 | <0.001 | 3A |
|  |  |  |  |  |  |  |
| WT | 17.077±0.308 | 17 | 22 | 214/216 | - | 3A |
| *smf-1* | 23.117±0.595 | 24 | 29 | 120/121 | <0.001 | 3A |
|  |  |  |  |  |  |  |
| WT | 17.077±0.308 | 17 | 22 | 214/216 | - | 3A |
| *smf-2* | 18.819±0.543 | 18 | 25 | 169/175 | <0.001 | 3A |
|  |  |  |  |  |  |  |
| WT | 17.077±0.308 | 17 | 22 | 214/216 | - | 3A |
| *smf-3* | 23.052±0.381 | 23 | 26 | 124/132 | <0.001 | 3A |
|  |  |  |  |  |  |  |
| WT | 17.404±0.347 | 17 | 22 | 174/176 | - | 3B |
| *fpn-1.1* | 19.740±0.410 | 21 | 21 | 25/44 | 0.727 | 3B |
|  |  |  |  |  |  |  |
| WT | 17.404±0.347 | 17 | 22 | 174/176 | - | 3B |
| *fpn-1.2* | 21.034±0.481 | 19 | 26 | 127/130 | <0.001 | 3B |
|  |  |  |  |  |  |  |
| WT | 17.404±0.347 | 17 | 22 | 174/176 | - | 3B |
| *F55H2.5* | 19.690±0.455 | 20 | 22 | 115/118 | <0.001 | 3B |
|  |  |  |  |  |  |  |
| WT | 17.404±0.347 | 17 | 22 | 174/176 | - | 3B |
| *mfn-1* | 20.069±0.387 | 19 | 24 | 144/150 | <0.001 | 3B |
|  |  |  |  |  |  |  |
| WT | 17.404±0.347 | 17 | 22 | 174/176 | - | 3B |
| *gcp-2.1* | 24.033±0.519 | 25 | 27 | 120/121 | <0.001 | 3B |
|  |  |  |  |  |  |  |
| WT | 17.404±0.347 | 17 | 22 | 174/176 | - | 3C |
| *hrg-4* | 21.966±0.710 | 23 | 28 | 142/148 | <0.001 | 3C |
|  |  |  |  |  |  |  |
| WT | 17.404±0.347 | 17 | 22 | 174/176 | - | 3C |
| *mrp-5* | 14.550±0.522 | 14 | 16 | 80/81 | <0.001 | 3C |
|  |  |  |  |  |  |  |
| WT | 17.228±0.506 | 17 | 22 | 95/97 | - | 3D |
| *ftn-1* | 14.359±0.431 | 15 | 17 | 109/111 | <0.001 | 3D |
|  |  |  |  |  |  |  |
| WT | 17.228±0.506 | 17 | 22 | 95/97 | - | 3D |
| *ftn-2* | 22.065±0.716 | 22 | 25 | 48/56 | <0.001 | 3D |
|  |  |  |  |  |  |  |
| WT | 23.668±0.536 | 26 | 28 | 125/148 | - | exp. 2 |
| *zipt-16* | 17.664±0.435 | 17 | 22 | 131/132 | <0.001 | exp. 2 |
|  |  |  |  |  |  |  |
| WT | 23.668±0.536 | 26 | 28 | 125/148 | - | exp. 2 |
| *zipt-17* | 15.483±0.319 | 15 | 19 | 121/122 | <0.001 | exp. 2 |
|  |  |  |  |  |  |  |
| WT | 23.668±0.536 | 26 | 28 | 125/148 | - | exp. 2 |
| *smf-1* | 23.117±0.595 | 24 | 29 | 120/121 | 0.666 | exp. 2 |
|  |  |  |  |  |  |  |
| WT | 23.668±0.536 | 26 | 28 | 125/148 | - | exp. 2 |
| *smf-2* | 18.690±0.490 | 18 | 25 | 217/228 | <0.001 | exp. 2 |
|  |  |  |  |  |  |  |
| WT | 23.668±0.536 | 26 | 28 | 125/148 | - | exp. 2 |
| *smf-3* | 22.929±0.393 | 23 | 26 | 119/127 | 0.023 | exp. 2 |
|  |  |  |  |  |  |  |
| WT | 23.668±0.536 | 26 | 28 | 125/148 | - | exp. 2 |
| *fpn-1.1* | 19.579±0.240 | 21 | 21 | 77/129 | <0.001 | exp. 2 |
|  |  |  |  |  |  |  |
| WT | 23.668±0.536 | 26 | 28 | 125/148 | - | exp. 2 |
| *fpn-1.2* | 21.142±0.480 | 19 | 26 | 129/132 | <0.001 | exp. 2 |
|  |  |  |  |  |  |  |
| WT | 23.668±0.536 | 26 | 28 | 125/148 | - | exp. 2 |
| *F55H2.5* | 18.992±0.493 | 19 | 24 | 119/120 | <0.001 | exp. 2 |
|  |  |  |  |  |  |  |
| WT | 23.668±0.536 | 26 | 28 | 125/148 | - | exp. 3 |
| *F55H2.5* | 19.490±0.362 | 20 | 22 | 185/198 | <0.001 | exp. 3 |
|  |  |  |  |  |  |  |
| WT | 23.668±0.536 | 26 | 28 | 125/148 | - | exp. 2 |
| *mfn-1* | 22.683±0.680 | 21 | 28 | 122/135 | 0.976 | exp. 2 |
|  |  |  |  |  |  |  |
| WT | 23.668±0.536 | 26 | 28 | 125/148 | - | exp. 2 |
| *gcp-2.1* | 24.033±0.519 | 25 | 27 | 120/121 | 0.709 | exp. 2 |
|  |  |  |  |  |  |  |
| WT | 23.668±0.536 | 26 | 28 | 125/148 | - | exp. 2 |
| *hrg-4* | 21.881±0.708 | 23 | 28 | 135/137 | 0.515 | exp. 2 |
|  |  |  |  |  |  |  |
| WT | 23.668±0.536 | 26 | 28 | 125/148 | - | exp. 2 |
| *mrp-5* | 15.405±0.639 | 14 | 16 | 84/85 | <0.001 | exp. 2 |
|  |  |  |  |  |  |  |
| WT | 23.668±0.536 | 26 | 28 | 125/148 | - | exp. 2 |
| *ftn-1* | 18.436±0.443 | 16 | 21 | 123/135 | <0.001 | exp. 2 |
|  |  |  |  |  |  |  |
| WT | 23.668±0.536 | 26 | 28 | 125/148 | - | exp. 2 |
| *ftn-2* | 22.536±0.425 | 22 | 25 | 151/165 | 0.022 | exp. 2 |

**Table 4: Statistics of phenotypic assays with various *C. elegans* iron homeostasis mutants.**

| **Strain** | **Mean** | ***P* value (mutant vs control)** | ***P* value (Day 7 vs Day 1)** | **Fig.** |
| --- | --- | --- | --- | --- |
| N2 (WT)/day 1 | 12.29 | - | - | 2A-2D |
| N2 (WT)/day 7 | 29.13 | - | 3.03364E-10 | 2A-2D |
| *smf-1*/day 1 | 7.323 | 1.03035E-10 | - | 2A |
| *smf-1*/day 7 | 22.08 | 0.000462 | 8.89706E-18 | 2A |
| *smf-2*/day 1 | 16.20 | 0.013555 | - | 2A |
| *smf-2*/day 7 | 33.33 | 0.078977 | 2.73597E-09 | 2A |
| *smf-3*/day 1 | 29.60 | 1.11667E-08 | - | 2A |
| *smf-3*/day 7 | 34.99 | 0.026920 | 0.061014942 | 2A |
| *zip16-1*/day 1 | 16.66 | 3.04214E-08 | - | 2A |
| *zip-16*/day 7 | 9.71 | 6.57639E-14 | 1.89098E-14 | 2A |
| *zip17-1*/day 1 | 18.078 | 3.54527E-12 | - | 2A |
| *zip-17*/day 7 | 11.36 | 7.41524E-13 | 3.69552E-16 | 2A |
|  |  |  |  |  |
| *fpn-1.1*/day 1 | 5.29 | 1.07211E-16 | - | 2B |
| *fpn-1.1*/day 7 | 16.91 | 1.49589E-08 | 4.8242E-16 | 2B |
| *fpn-1.2*/day 1 | 11.21 | 0.059318753 | - | 2B |
| *fpn-1.2*/day 7 | 17.58 | 2.02365E-08 | 2.47742E-11 | 2B |
| *F55H2.5*/day 1 | 22.60 | 5.10E-09 | - | 2B |
| *F55H2.5*/day 7 | 23.53 | 0.002378921 | 0.423393544 | 2B |
| *mfn-1*/day 1 | 5.95 | 3.51957E-15 | - | 2B |
| *mfn-1*/day 7 | 40.58 | 1.10806E-06 | 3.96144E-19 | 2B |
| *gcp-2.1*/day 1 | 36.01 | 6.88912E-12 | - | 2B |
| *gcp-2.1*/day 7 | 47.83 | 6.31339E-06 | 0.004187106 | 2B |
|  |  |  |  |  |
| *hrg-4*/day 1 | 13.88 | 0.045445604 | - | 2C |
| *hrg-4*/day 7 | 15.59 | 1.06239E-09 | 0.042690082 | 2C |
| *mrp-5*/day 1 | 8.18 | 1.12441E-09 | - | 2C |
| *mrp-5*/day 7 | 10.57 | 2.06225E-13 | 2.25255E-07 | 2C |
|  |  |  |  |  |
| *ftn-1*/day 1 | 9.98 | 0.017957747 | - | 2D |
| *ftn-1*/day 7 | 8.69 | 4.56942E-15 | 0.207140702 | 2D |
| *ftn-2*/day 1 | 18.43 | 7.75825E-12 | - | 2D |
| *ftn-2*/day 7 | 18.75 | 6.43707E-07 | 0.709331386 | 2D |
|  |  |  |  |  |
| N2 (WT)/day 1 | 216.57 | - | - | 4A-4D |
| N2 (WT)/day 7 | 116.28 | - | 3.78886E-05 | 4A-4D |
| *smf-1*/day 1 | 183.00 | 0.164018533 | - | 4A |
| *smf-1*/day 7 | 98.75 | 0.207878056 | 0.000945112 | 4A |
| *smf-2*/day 1 | 137.21 | 0.003329684 | - | 4A |
| *smf-2*/day 7 | 54.91 | 1.54779E-07 | 0.000800086 | 4A |
| *smf-3*/day 1 | 125.51 | 0.001183183 | - | 4A |
| *smf-3*/day 7 | 114.14 | 0.890522918 | 0.637709556 | 4A |
| *zip16-1*/day 1 | 138.36 | 0.394517075 | - | 4A |
| *zip-16*/day 7 | 115.83 | 0.922033448 | 0.15309484 | 4A |
| *zip17-1*/day 1 | 177.23 | 0.027587894 | - | 4A |
| *zip-17*/day 7 | 77.92 | 0.041604959 | 2.27668E-07 | 4A |
|  |  |  |  |  |
| *fpn-1.1*/day 1 | 163.35 | 0.068034776 | - | 4B |
| *fpn-1.1*/day 7 | 105.98 | 0.361432974 | 0.067584618 | 4B |
| *fpn-1.2*/day 1 | 223.59 | 0.930431303 | - | 4B |
| *fpn-1.2*/day 7 | 48.68 | 9.5907E-08 | 3.77774E-12 | 4B |
| *F55H2.5*/day 1 | 114.09 | 2.11055E-05 | - | 4B |
| *F55H2.5*/day 7 | 55.17 | 6.98433E-07 | 0.000353201 | 4B |
| *mfn-1*/day 1 | 176.88 | 0.089931187 | - | 4B |
| *mfn-1*/day 7 | 105.12 | 0.279219233 | 0.000627708 | 4B |
| *gcp-2.1*/day 1 | 161.47 | 0.033439846 | - | 4B |
| *gcp-2.1*/day 7 | 96.11 | 0.092198546 | 0.009371993 | 4B |
|  |  |  |  |  |
| *hrg-4*/day 1 | 178.43 | 0.07321313 | - | 4C |
| *hrg-4*/day 7 | 175.59 | 0.000149959 | 0.869244716 | 4C |
| *mrp-5*/day 1 | 171.63 | 0.140282256 | - | 4C |
| *mrp-5*/day 7 | 87.91 | 0.003958105 | 0.005938683 | 4C |
|  |  |  |  |  |
| *ftn-1*/day 1 | 139.31 | 0.00056808 | - | 4D |
| *ftn-1*/day 7 | 19.31 | 5.6427E-07 | 2.26707E-08 | 4D |
| *ftn-2*/day 1 | 147.98 | 0.017296025 | - | 4D |
| *ftn-2*/day 7 | 79.27 | 0.00072562 | 0.009605739 | 4D |
|  |  |  |  |  |
| N2 (WT)/day 1 | 1028.23 | - | - | 5A-5D |
| N2 (WT)/day 7 | 1354.33 | - | 3.15577E-14 | 5A-5D |
| *smf-1*/day 1 | 818.07 | 5.17304E-10 | - | 5A |
| *smf-1*/day 7 | 1082.78 | 3.12008E-12 | 1.43302E-11 | 5A |
| *smf-2*/day 1 | 996.69 | 0.055522687 | - | 5A |
| *smf-2*/day 7 | 1167.67 | 0.00015393 | 0.00035623 | 5A |
| *smf-3*/day 1 | 860.80 | 1.81382E-09 | - | 5A |
| *smf-3*/day 7 | 1174.52 | 7.80752E-06 | 7.09544E-09 | 5A |
| *zip16-1*/day 1 | 753.81 | 0.002983392 | - | 5A |
| *zip-16*/day 7 | 1379.27 | 2.6985E-06 | 7.12335E-15 | 5A |
| *zip17-1*/day 1 | 1021.08 | 0.64952351 | - | 5A |
| *zip-17*/day 7 | 1256.35 | 0.016007177 | 6.64271E-10 | 5A |
|  |  |  |  |  |
| *fpn-1.1*/day 1 | 996.59 | 0.925693316 | - | 5B |
| *fpn-1.1*/day 7 | 1104.71 | 2.57423E-12 | 0.067692411 | 5B |
| *fpn-1.2*/day 1 | 1090.54 | 0.004668061 | - | 5B |
| *fpn-1.2*/day 7 | 1397.03 | 0.1401247 | 4.85006E-08 | 5B |
| *F55H2.5*/day 1 | 972.45 | 0.306791719 | - | 5B |
| *F55H2.5*/day 7 | 1207.30 | 3.31713E-05 | 0.000214012 | 5B |
| *mfn-1*/day 1 | 1007.55 | 0.2166934 | - | 5B |
| *mfn-1*/day 7 | 1375.65 | 0.465297402 | 5.2973E-15 | 5B |
| *gcp-2.1*/day 1 | 907.63 | 0.001139925 | - | 5B |
| *gcp-2.1*/day 7 | 1295.85 | 0.020002884 | 5.68332E-10 | 5B |
|  |  |  |  |  |
| *hrg-4*/day 1 | 966.72 | 0.071794356 | - | 5C |
| *hrg-4*/day 7 | 1155.14 | 1.01892E-10 | 4.30925E-05 | 5C |
| *mrp-5*/day 1 | 1003.53 | 0.24483207 | - | 5C |
| *mrp-5*/day 7 | 1174.86 | 7.47898E-05 | 0.000129335 | 5C |
|  |  |  |  |  |
| *ftn-1*/day 1 | 920.13 | 5.86089E-05 | - | 5D |
| *ftn-1*/day 7 | 1253.36 | 0.000212438 | 1.29885E-10 | 5D |
| *ftn-2*/day 1 | 812.15 | 0.001062334 | - | 5D |
| *ftn-2*/day 7 | 1291.38 | 0.009380103 | 1.81411E-05 | 5D |
|  |  |  |  |  |
| N2 (WT)/day 1 | 60.24 | - | - | 5E-5H |
| N2 (WT)/day 7 | 84.06 | - | 4.14292E-13 | 5E-5H |
| *smf-1*/day 1 | 58.40 | 0.319838668 | - | 5E |
| *smf-1*/day 7 | 62.76 | 1.14506E-11 | 0.046501704 | 5E |
| *smf-2*/day 1 | 76.27 | 0.000812408 | - | 5E |
| *smf-2*/day 7 | 91.80 | 0.000416247 | 0.001104536 | 5E |
| *smf-3*/day 1 | 61.82 | 0.417256866 | - | 5E |
| *smf-3*/day 7 | 78.54 | 0.041664583 | 0.009083897 | 5E |
| *zip16-1*/day 1 | 48.60 | 2.42745E-05 | - | 5E |
| *zip-16*/day 7 | 84.93 | 4.24136E-05 | 4.02533E-12 | 5E |
| *zip17-1*/day 1 | 72.71 | 0.000508176 | - | 5E |
| *zip-17*/day 7 | 86.58 | 0.003180931 | 1.01379E-08 | 5E |
|  |  |  |  |  |
| *fpn-1.1*/day 1 | 66.64 | 0.162363651 | - | 5F |
| *fpn-1.1*/day 7 | 71.16 | 2.04914E-07 | 0.101740881 | 5F |
| *fpn-1.2*/day 1 | 78.57 | 8.53888E-08 | - | 5F |
| *fpn-1.2*/day 7 | 78.92 | 0.008603014 | 0.702011707 | 5F |
| *F55H2.5*/day 1 | 58.58 | 0.517816909 | - | 5F |
| *F55H2.5*/day 7 | 78.23 | 0.064756677 | 6.94861E-07 | 5F |
| *mfn-1*/day 1 | 66.33 | 0.000975344 | - | 5F |
| *mfn-1*/day 7 | 84.98 | 0.480432014 | 5.16847E-12 | 5F |
| *gcp-2.1*/day 1 | 75.12 | 0.000105514 | - | 5F |
| *gcp-2.1*/day 7 | 87.31 | 0.032548015 | 0.000214797 | 5F |
|  |  |  |  |  |
| *hrg-4*/day 1 | 64.09 | 0.074049145 | - | 5G |
| *hrg-4*/day 7 | 72.63 | 2.54453E-07 | 0.000777775 | 5G |
| *mrp-5*/day 1 | 78.07 | 3.87467E-05 | - | 5G |
| *mrp-5*/day 7 | 86.10 | 0.288717502 | 0.023554224 | 5G |
|  |  |  |  |  |
| *ftn-1*/day 1 | 66.28 | 0.000934213 | - | 5H |
| *ftn-1*/day 7 | 77.04 | 0.001370248 | 6.71014E-06 | 5H |
| *ftn-2*/day 1 | 59.28 | 0.643835339 | - | 5H |
| *ftn-2*/day 7 | 86.35 | 0.09054158 | 0.40931026 | 5H |
|  |  |  |  |  |
| N2 (WT)/day 1 | 100 | - | - | 6A-6D |
| N2 (WT)/day 7 | 83.33 | - | 0.0075 | 6A-6D |
| *smf-1*/day 1 | 100 | - | - | 6A |
| *smf-1*/day 7 | 86.19 | 0.5656693 | 0.047515 | 6A |
| *smf-2*/day 1 | 98.095 | 0.3910022 | - | 6A |
| *smf-2*/day 7 | 73.33 | 0.1011915 | 0.0063146 | 6A |
| *smf-3*/day 1 | 100 | - | - | 6A |
| *smf-3*/day 7 | 70 | 0.1335832 | 0.0350987 | 6A |
| *zip16-1*/day 1 | 98.89 | 0.4226497 | - | 6A |
| *zip-16*/day 7 | 84.29 | 0.8417237 | 0.02734 | 6A |
| *zip17-1*/day 1 | 100 | - | - | 6A |
| *zip-17*/day 7 | 90 | - | 0.2254033 | 6A |
|  |  |  |  |  |
| *fpn-1.1*/day 1 | 99.05 | 0.42265 | - | 6B |
| *fpn-1.1*/day 7 | 57.78 | 0.034465 | 0.019746 | 6B |
| *fpn-1.2*/day 1 | 98.10 | 0.42265 | - | 6B |
| *fpn-1.2*/day 7 | 80.32 | 0.461 | 0.009741 | 6B |
| *F55H2.5*/day 1 | 100 | - | - | 6B |
| *F55H2.5*/day 7 | 26.85 | 0.000296 | 0.002191 | 6B |
| *mfn-1*/day 1 | 91.90 | 0.195928 | - | 6B |
| *mfn-1*/day 7 | 57.78 | 0.004892 | 0.005506 | 6B |
| *gcp-2.1*/day 1 | 93.81 | 0.185908 |  | 6B |
| *gcp-2.1*/day 7 | 52.86 | 0.052646 | 0.027603 | 6B |
|  |  |  |  |  |
| *hrg-4*/day 1 | 98.10 | 0.42265 | - | 6C |
| *hrg-4*/day 7 | 52.86 | 0.003474 | 0.001465 | 6C |
| *mrp-5*/day 1 | 100 | - | - | 6C |
| *mrp-5*/day 7 | 93.33 | 0.101192 | 0.183503 | 6C |
|  |  |  |  |  |
| *ftn-1*/day 1 | 99.05 | 0.42265 | - | 6D |
| *ftn-1*/day 7 | 86.32 | 0.725973 | 0.207654 | 6D |
| *ftn-2*/day 1 | 100 | - | - | 6D |
| *ftn-2*/day 7 | 65.24 | 0.015476 | 0.006872 | 6D |
